# Antibody-Stratified FNA-Thyroglobulin Cut-Off Values for Preoperative Lymph Node Assessment in Differentiated Thyroid Cancer

**DOI:** 10.3390/diagnostics16091344

**Published:** 2026-04-29

**Authors:** Beril Turan Erdogan, Kubra Durmus Demirel, Fatma Dilek Dellal Kahramanca, Fazli Erdogan, Yunus Nadi Yuksek, Cevdet Aydin, Oya Topaloglu, Reyhan Ersoy, Bekir Cakir

**Affiliations:** 1 Department of Endocrinology and Metabolism, Ankara Bilkent City Hospital, 06800 Ankara, Turkey; 2 Department of Pathology, Faculty of Medicine, Ankara Yildirim Beyazit University, 06800 Ankara, Turkey; 3 Department of General Surgery, Ankara Bilkent City Hospital, 06800 Ankara, Turkey; 4 Department of Endocrinology and Metabolism, Faculty of Medicine, Ankara Yildirim Beyazit University, 06800 Ankara, Turkey

**Keywords:** differentiated thyroid cancer, fine-needle aspiration, thyroglobulin, anti-thyroglobulin antibodies, lymph node metastasis, preoperative diagnosis

## Abstract

**Background:** Fine-needle aspiration thyroglobulin (FNA-Tg) is widely used to detect lymph node metastases in differentiated thyroid cancer (DTC), but optimal cut-off values remain controversial. Anti-thyroglobulin antibodies (anti-Tg), present in 25–40% of DTC patients, may interfere with FNA-Tg measurements. This study aimed to evaluate whether anti-Tg status necessitates different FNA-Tg diagnostic thresholds in the preoperative setting. **Methods:** We retrospectively analyzed 605 cervical lymph nodes from 393 preoperative DTC patients who underwent ultrasound-guided fine-needle aspiration (FNA) with concurrent FNA-Tg measurement (February 2019–April 2025). All lymph nodes had histopathological or cytological confirmation. Patients were stratified by anti-Tg status (>4.5 IU/mL). Receiver operating characteristic curve analysis determined optimal FNA-Tg cut-offs, and areas under the curve (AUCs) were compared using the DeLong test. **Results:** FNA-Tg demonstrated excellent overall diagnostic accuracy (AUC 0.963, 95% CI 0.946–0.980) with an optimal cut-off of 84.0 ng/mL (sensitivity 91.6%, specificity 95.3%). Anti-Tg-positive patients had significantly lower FNA-Tg levels in malignant lymph nodes compared to anti-Tg-negative patients (median 9872 vs. 22,327 ng/mL, *p* = 0.001). Subgroup analysis revealed superior performance in anti-Tg-negative patients (AUC 0.983, cut-off 84.4 ng/mL) compared to anti-Tg-positive patients (AUC 0.923, cut-off 65.7 ng/mL; *p* = 0.008). No significant correlation was observed between anti-Tg levels and FNA-Tg (ρ = −0.03, *p* = 0.501). **Conclusions:** Anti-Tg status influenced measured FNA-Tg levels and receiver operating characteristic (ROC) derived optimal thresholds in the preoperative setting. However, because malignant lymph nodes generally showed FNA-Tg values well above the benign range, the clinical impact of this difference appears limited in most clearly positive cases. These findings may still help refine interpretation in selected borderline cases.

## 1. Introduction

Differentiated thyroid cancer (DTC) accounts for over 90% of all thyroid malignancies and generally carries a favorable prognosis. However, cervical lymph node metastasis occurs in 20–50% of patients at initial diagnosis and represents a significant risk factor for locoregional recurrence and disease-related mortality [1,2]. Accurate preoperative identification of metastatic lymph nodes is essential for surgical planning, as it determines the extent of lymph node dissection and influences long-term outcomes. While ultrasound imaging has improved the detection of suspicious lymph nodes, fine-needle aspiration cytology (FNAC) remains limited by sampling errors and low cellularity, particularly in cystic or necrotic nodes, leading to false-negative rates of 20–30% [3,4].

Measurement of thyroglobulin in fine-needle aspiration (FNA-Tg) has emerged as a valuable adjunct to cytology for detecting lymph node metastases in DTC patients. Since its introduction three decades ago, FNA-Tg has demonstrated superior sensitivity compared to cytology alone, with reported accuracies exceeding 90% in most studies [5,6]. The combination of FNA-Tg and cytology significantly improves diagnostic performance, reducing false-negative results and enhancing clinical decision-making. Despite its widespread adoption, several factors complicate the interpretation of FNA-Tg results, including the presence of thyroid tissue, serum TSH levels, and most notably, circulating anti-thyroglobulin antibodies (anti-Tg).

Anti-thyroglobulin antibodies, present in approximately 25–40% of DTC patients, can interfere with serum thyroglobulin (Tg) measurements via immunoassay-based mechanisms. However, the impact of anti-Tg on FNA-Tg levels remains controversial, with conflicting reports in the literature [2,7]. While some studies suggest minimal interference in the washout fluid due to high local Tg concentrations, others have documented lower FNA-Tg values in anti-Tg-positive patients with proven metastases. Furthermore, the optimal cut-off value for FNA-Tg varies widely across studies, ranging from 1 to 100 ng/mL, and no consensus exists on whether anti-Tg status should influence threshold selection in the preoperative setting [8].

Given these uncertainties, we conducted this study to evaluate the diagnostic performance of FNA-Tg in preoperative DTC patients and to determine whether anti-Tg status necessitates different cut-off values. We analyzed a large cohort of lymph nodes with histopathological correlation, comparing the diagnostic accuracy of FNA-Tg between anti-Tg-negative and anti-Tg-positive patients. Our goal was to evaluate whether subgroup-specific thresholds could improve the preoperative assessment of cervical lymph nodes in DTC.

## 2. Materials and Methods

### 2.1. Study Design and Setting

This retrospective observational study was conducted at the Department of Endocrinology and Metabolism of Ankara Bilkent City Hospital, a tertiary referral center for thyroid cancer management. The study included patients with histologically confirmed differentiated thyroid cancer who underwent preoperative ultrasound-guided fine-needle aspiration (FNA) of cervical lymph nodes with concurrent FNA-Tg measurement between February 2019 and April 2025.

Ankara Bilkent City Hospital ethical committee approval was obtained on 04 June 2025 (TABED 1-25-1367) in accordance with the ethical standards of the Declaration of Helsinki.

### 2.2. Study Population

Patients with differentiated thyroid cancer scheduled for thyroidectomy and cervical lymph node dissection were eligible for inclusion. Between February 2019 and April 2025, a total of 605 cervical lymph nodes from 393 patients underwent ultrasound-guided FNA with simultaneous FNA-Tg measurement and were included in the final analytic dataset. More than one lymph node could be included from the same patient when multiple sonographically suspicious nodes were sampled.

Lymph nodes lacking definitive histopathological characterization following surgery were excluded. Patients with incomplete biochemical data, missing anti-Tg measurements, or insufficient imaging documentation were also excluded. The final cohort consisted of preoperative DTC patients with both complete FNA-Tg measurements and surgical pathology confirmation of lymph node status.

### 2.3. Ultrasound Evaluation and Fine-Needle Aspiration Procedure

All cervical lymph nodes were evaluated by experienced endocrinologists using high-resolution ultrasonography with a 7.5–12 MHz linear transducer on an Aplio 500 ultrasound system (Toshiba Medical Systems Corporation, Otawara, Tochigi, Japan). At our institution, preoperative US-guided FNA of cervical lymph nodes was performed selectively in sonographically suspicious nodes when cytological and/or Tg-washout confirmation was considered relevant to surgical planning, particularly the extent of nodal dissection. When multiple suspicious lymph nodes were present in the same patient, more than one node could be sampled at the discretion of the treating clinicians based on ultrasonographic suspicion and anticipated relevance for surgical planning. Because of the retrospective design, the specific management-related rationale for biopsy was not uniformly documented for every case. Nodes were considered suspicious if they demonstrated one or more ultrasonographic features associated with malignant involvement—loss of hilum, cystic change, microcalcifications, marked hypoechogenicity, round morphology (Solbiati index < 2), or peripheral/chaotic vascularity—as defined in the 2015 American Thyroid Association (ATA) Guidelines [9].

Ultrasound-guided fine-needle aspiration was performed using 24–27 gauge needles under real-time sonographic guidance. After advancing the needle into the lymph node, 2–4 passes with multiple short excursions were performed until sufficient material accumulated within the needle hub. The aspirated material was expelled onto glass slides for cytological assessment. Immediately thereafter, the same needle and syringe were flushed with exactly 0.6 mL of sterile normal saline, and the washout fluid was collected for thyroglobulin analysis. Standardizing the washout volume and using the same needle and syringe were intended to reduce procedural variability and minimize potential contamination.

Concurrent serum thyroglobulin was obtained at the time of FNA. All washout and serum samples were delivered promptly to the biochemistry laboratory for analysis.

### 2.4. Laboratory Measurements

Serum thyroglobulin, anti-thyroglobulin antibody, thyroid-stimulating hormone, free thyroxine, and free triiodothyronine concentrations, as well as thyroglobulin levels in fine-needle aspiration washout fluid, were measured using a chemiluminescent immunometric assay (Siemens Immulite 2000, Siemens Healthineers Tarrytown, NY, USA).

According to the manufacturer’s specifications, the reference ranges were 0.55–4.78 mIU/L for TSH, 0.89–1.76 ng/dL for free T4, and 2.3–4.2 pg/mL for free T3. Anti-Tg positivity was defined as >4.5 IU/mL based on the manufacturer’s threshold for autoimmune thyroiditis, ensuring identification of clinically significant antibody interference.

### 2.5. Reference Standard for Lymph Node Classification

Lymph node classification was determined hierarchically: (1) cytology diagnostic of malignancy → classified as malignant; (2) benign cytology → classified as benign; (3) nondiagnostic cytology with elevated FNA-Tg → classified based on histopathological confirmation when available. Lymph nodes lacking definitive cytological or histopathological characterization, or aspirates consistent with residual thyroid tissue, postoperative lymphocele, or other non-nodal structures, were excluded from analysis. This exclusion was intended to reduce misclassification and to minimize the potential influence of non-nodal or thyroidal contamination on FNA-Tg interpretation.

### 2.6. Statistical Analysis

Statistical analyses were performed using Python 3.10 scientific libraries and SPSS version 26.0 (IBM Corp., Armonk, NY, USA). Continuous variables were expressed as median and interquartile range (IQR) due to non-normal distribution (Kolmogorov–Smirnov test). Group comparisons between benign and malignant lymph nodes were conducted using the Mann–Whitney U test. Categorical variables were compared using the χ^2^ or Fisher’s exact test, as appropriate.

The relationships between FNA-Tg, serum thyroglobulin, and anti-thyroglobulin antibody levels were assessed with Spearman’s rank correlation coefficients. For FNA-Tg measurements below the assay limit of detection (LoD), values were imputed using LoD/√2, corresponding to 0.14 ng/mL for measurements < 0.2 ng/mL, a standard approach for handling left-censored data in biomarker studies. The primary unit of analysis was the lymph node. The diagnostic accuracy of FNA-Tg for detecting lymph node metastasis was evaluated by receiver operating characteristic (ROC) curve analysis, and the area under the curve (AUC) was calculated with 95% confidence intervals. Optimal cut-off values were determined by maximizing the Youden index (sensitivity + specificity − 1).

To evaluate whether anti-Tg status modified the diagnostic performance of FNA-Tg, ROC analyses were repeated in anti-Tg-negative and anti-Tg-positive subgroups. The AUCs between groups were compared using the DeLong method. Because multiple lymph nodes could belong to the same patient, sensitivity analyses were also performed with cluster-robust variance estimators using a patient-level random intercept. Statistical significance was defined as *p* < 0.05 (two-tailed).

## 3. Results

A total of 605 lymph node lesions from 393 patients (median age, 46 years; 74.3% female) were analyzed. Histopathological examination revealed malignancy in 225 (37.2%) lymph nodes. Anti-Tg positivity was present in 219 lesions (36.2%), and thyroid peroxidase antibodies (anti-TPO) were positive in approximately 41% of patients. The baseline demographic and clinical characteristics of the study cohort are summarized in Table 1.

When benign and malignant lymph nodes were compared, malignant nodes demonstrated significantly larger dimensions in all planes. Median anteroposterior, transverse, and longitudinal diameters were 7.5, 10.8, and 12.2 mm, respectively, compared with 5.0, 7.0, and 8.8 mm in benign nodes (*p* < 0.001 for all, Table 2). Serum thyroglobulin (Tg) levels tended to be higher in malignant lesions (46.0 ng/mL [IQR 12.0–140.0]) than in benign lesions (31.8 ng/mL [8.4–88.4]), although the difference did not reach statistical significance (*p* = 0.076). In contrast, Tg-washout concentrations were markedly higher in malignant nodes (median 16,141 ng/mL [IQR 1883–30,000]) than in benign nodes (2.01 ng/mL [0.54–6.65]; *p* < 0.001).

After stratification by anti-Tg status (Table 3), Tg-washout values remained significantly elevated in malignant lesions in both subgroups. However, malignant nodes of anti-Tg–positive patients exhibited lower Tg-washout levels (median 9872 ng/mL [IQR 374–30,000]) than those of anti-Tg–negative patients (22,327 ng/mL [IQR 2450–30,000]; *p* = 0.001). Among benign nodes, Tg-washout levels were comparable between anti-Tg-negative and -positive patients (*p* = 0.25). Serum Tg concentrations were inversely associated with anti-Tg positivity.

Correlation analysis demonstrated a moderate positive association between Tg-washout and serum Tg (ρ = 0.261, *p* < 0.001) and a strong inverse correlation between anti-Tg and serum Tg (ρ = −0.496, *p* < 0.001), whereas no significant relationship was observed between anti-Tg and Tg-washout (ρ = −0.03, *p* = 0.501) (Table 4).

**Figure 1:**
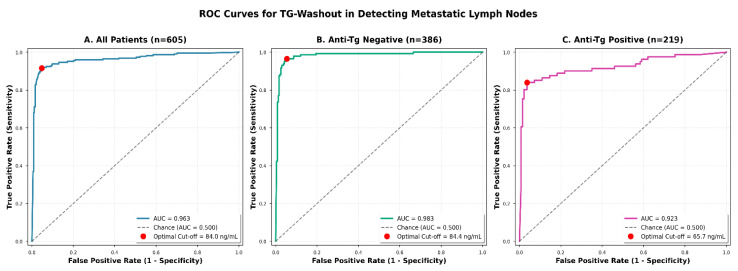
ROC curves for Tg-washout in detecting metastatic lymph nodes. ROC curve analysis demonstrating the diagnostic performance of fine-needle aspiration thyroglobulin washout (Tg-washout) in distinguishing metastatic from benign cervical lymph nodes in preoperative differentiated thyroid cancer patients. Panel (**A**) shows the ROC curve for all patients (*n* = 605), achieving an area under the curve (AUC) of 0.963 with an optimal cut-off value of 84.0 ng/mL determined by the Youden index. Panel (**B**) displays the ROC curve for patients with negative anti-thyroglobulin antibodies (anti-Tg negative, *n* = 386), demonstrating superior diagnostic performance with an AUC of 0.983 and an optimal cut-off of 84.4 ng/mL. Panel (**C**) presents the ROC curve for patients with positive anti-thyroglobulin antibodies (anti-Tg positive, *n* = 219), showing an AUC of 0.923 with a lower optimal cut-off of 65.7 ng/mL. The red dots indicate the optimal cut-off points determined by the Youden index (J = sensitivity + specificity − 1). The diagonal dashed line represents the chance line (AUC = 0.500). The difference in AUCs between anti-Tg-negative and -positive groups was statistically significant (*p* = 0.008, DeLong test), indicating that anti-thyroglobulin antibody status significantly impacts the diagnostic performance of Tg-washout. AUC: area under the curve; Anti-Tg: anti-thyroglobulin antibody; ROC: receiver operating characteristic.

**Figure 2:**
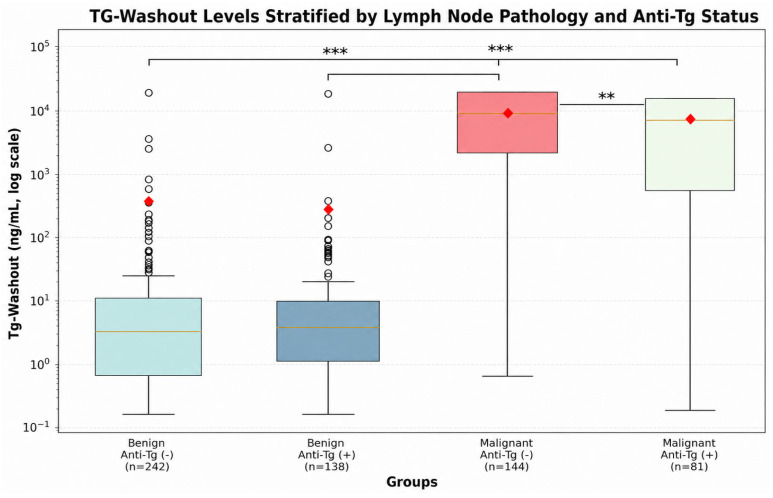
Tg-washout levels stratified by lymph node pathology and anti-thyroglobulin antibody status. Box plot comparison of fine-needle aspiration thyroglobulin washout (Tg-washout) levels across four groups stratified by lymph node pathology and anti-thyroglobulin antibody status: benign/anti-Tg negative (*n* = 242), benign/anti-Tg positive (*n* = 138), malignant/anti-Tg negative (*n* = 144), and malignant/anti-Tg positive (*n* = 81). Tg-washout values are shown on a logarithmic scale. Box plots indicate the median, interquartile range, whiskers, and outliers; red diamonds indicate group means. Horizontal lines denote pairwise comparisons using the Mann–Whitney U test. *** *p* < 0.001 and ** *p* < 0.01. Anti-Tg, anti-thyroglobulin antibody; Tg, thyroglobulin. ROC: receiver operating characteristic.

ROC curve analysis showed that Tg-washout had excellent diagnostic accuracy for identifying metastatic lymph nodes, with an AUC of 0.963 (95% CI 0.946–0.980; Figure 1). The optimal cut-off value determined by the Youden index was 84.0 ng/mL, yielding 91.6% sensitivity, 95.3% specificity, and 93.9% overall accuracy. In subgroup analyses (Table 5, Figure 1), AUC values were 0.983 in anti-Tg-negative and 0.923 in anti-Tg-positive patients, with optimal cut-offs of 84.4 ng/mL and 65.7 ng/mL, respectively. The difference between AUCs was statistically significant (*p* = 0.008, DeLong test). The log-scaled distribution of Tg-washout levels across benign and malignant lesions according to anti-Tg status is illustrated in Figure 2.

## 4. Discussion

In this large preoperative cohort, FNA-Tg showed excellent diagnostic accuracy for identifying metastatic cervical lymph nodes in patients with differentiated thyroid cancer, with an overall AUC of 0.963. The principal finding of this study is that circulating anti-Tg antibodies were associated with lower Tg-washout levels in malignant lymph nodes and with a lower ROC-derived optimal threshold. However, because malignant lymph nodes generally showed values well above the benign range, the clinical magnitude of this effect appears limited in most clearly positive preoperative cases. Nevertheless, in selected borderline cases where Tg-washout values fall near the diagnostic threshold, anti-Tg status may warrant consideration when choosing an appropriate cut-off.

The overall diagnostic performance observed in our cohort is consistent with previous large-scale studies reporting high accuracy of FNA-Tg for detecting nodal metastases [5,6]. However, recent evidence suggests that the diagnostic characteristics of FNA-Tg differ substantially between preoperative and postoperative contexts. A 2025 systematic review and meta-analysis demonstrated superior sensitivity and specificity in postoperative patients compared with those evaluated preoperatively [10]. This distinction is clinically important and directly supports our observation that optimal cut-off values in the preoperative setting are considerably higher than those reported in postoperative surveillance studies. In most earlier investigations, patients had undergone thyroidectomy, often with radioactive iodine ablation, effectively eliminating baseline thyroglobulin production [7,11]. In contrast, all patients in our cohort had intact thyroid tissue, resulting in ongoing thyroidal thyroglobulin secretion and elevated baseline FNA-Tg levels even in benign lymph nodes. This biological context fundamentally alters the interpretive framework of FNA-Tg and explains why thresholds derived from postoperative cohorts cannot be directly extrapolated to preoperative decision-making.

Within this preoperative environment, anti-thyroglobulin antibody status emerged as a critical modifier of FNA-Tg interpretation. Malignant lymph nodes from anti-Tg-positive patients exhibited substantially lower FNA-Tg concentrations than those from antibody-negative patients, despite histopathological confirmation of metastatic involvement in both groups. Importantly, this difference was observed only in malignant nodes, whereas benign lymph nodes showed similar FNA-Tg values regardless of antibody status. These findings suggest that anti-Tg interference becomes clinically relevant at the high thyroglobulin concentrations typical of metastatic disease, precisely where diagnostic discrimination is most critical.

This observation contrasts with the saturation hypothesis proposed in earlier studies, which suggested that extremely high local thyroglobulin concentrations would overwhelm any antibody-mediated interference [2]. Our data do not support this assumption. Instead, they indicate that antibody-related effects persist even at high FNA-Tg levels. Consistent with prior reports, we observed no significant correlation between serum anti-Tg titers and FNA-Tg concentrations [7,12], suggesting that interference may operate at the assay level rather than through a simple dose-dependent relationship. Alternative explanations include immune complex formation reducing the fraction of free thyroglobulin available for detection, a negative correlation between anti-Tg titers and FNA-Tg concentrations, or broader immunological differences influencing thyroglobulin secretion by metastatic cells [7,13,14,15,16]. At the same time, the extent and direction of anti-Tg interference cannot be predicted reliably in individual samples. For this reason, the lower cut-off identified in anti-Tg-positive patients should be regarded as a cohort-specific ROC-derived finding rather than a fixed threshold ready for routine clinical application. Although the underlying mechanism cannot be definitively established from our data, the practical implication is that anti-Tg status should be taken into account when interpreting FNA-Tg results in the preoperative setting.

The clinical implications of these findings should be interpreted with caution. In our cohort, malignant lymph nodes generally showed Tg-washout concentrations far above the benign range, regardless of anti-Tg status. Therefore, although anti-Tg positivity was associated with lower Tg-washout levels and a lower ROC-derived optimal threshold, the practical impact of this difference may be limited in most clearly positive preoperative cases. Rather than supporting a major change in routine practice, our findings suggest that anti-Tg status may be most relevant when interpreting borderline or equivocal Tg-washout results. This issue should be clarified in prospective studies specifically designed to assess the clinical utility of antibody-stratified thresholds.

Our findings are consistent with recent large retrospective studies identifying anti-Tg status as a significant factor influencing FNA-Tg measurements [16] and align with observations reported by Shin et al. and Jo et al., who also noted reduced FNA-Tg levels in antibody-positive patients with confirmed metastases [7,8]. Moreover, the dependence of optimal cut-off values on thyroid gland status has been highlighted in prior work examining mixed preoperative and postoperative populations [17]. Together, these data reinforce the concept that FNA-Tg interpretation must be contextualized according to both immunological status and the presence of residual thyroid tissue.

The updated 2025 American Thyroid Association guidelines emphasize the importance of accurate preoperative lymph node evaluation to guide surgical management [18]. Although our ultrasound criteria were based on the 2015 ATA recommendations, the principle that diagnostic thresholds should be adapted to clinical context remains highly relevant. Our results provide quantitative evidence supporting a more personalized approach to FNA-Tg interpretation in preoperative patients and may help refine guideline-based decision-making in this setting.

Several limitations of our study should be acknowledged. The retrospective design introduces selection bias, as lymph nodes were sampled based on sonographic features suggestive of malignancy, potentially overrepresenting high-risk lesions. In addition, although preoperative lymph node FNA was performed selectively in sonographically suspicious nodes, the specific management-related rationale for biopsy was not uniformly documented for each case because of the retrospective design. The single-center nature of the study limits generalizability, particularly given variability in assay platforms and institutional practices. Although we used a widely adopted chemiluminescent immunoassay, absolute FNA-Tg values and optimal thresholds may differ across analytical systems. In addition, while serum TSH levels varied among patients, ongoing thyroidal thyroglobulin secretion in this preoperative cohort appears to have exerted a greater influence on FNA-Tg levels than TSH-mediated stimulation. Because all patients were evaluated preoperatively with intact thyroid tissue, serum or thyroidal contamination of FNA washout samples cannot be completely excluded despite ultrasound-guided sampling, exclusion of non-nodal aspirates, and a standardized washout technique. This may have contributed to background FNA-Tg levels in benign lymph nodes and to the relatively higher cut-off values observed in the preoperative setting. Residual confounding from factors such as lymph node vascularity, necrosis, or cystic degeneration cannot be excluded. Finally, external validation in independent preoperative cohorts is required before widespread adoption of antibody-stratified cut-off values can be recommended. Additionally, we did not evaluate the impact of different anti-Tg assay platforms, which may vary in their degree of interference with thyroglobulin measurement and warrant platform-specific cut-off validation.

In conclusion, FNA-Tg showed excellent diagnostic accuracy for preoperative detection of lymph node metastases in DTC. Anti-Tg positivity was associated with lower Tg-washout values in malignant lymph nodes and with a lower ROC-derived optimal threshold. However, because malignant nodes in our cohort generally had Tg-washout concentrations well above the benign range, the clinical impact of this difference appears limited in most routine preoperative cases. Anti-Tg status may still be relevant when interpreting borderline results, but the clinical value of antibody-stratified thresholds should be confirmed in prospective studies.

## Figures and Tables

**Table 1:** Baseline demographic, clinical, and surgical characteristics of patients.

Variable	Total (*n* = 393)	Benign (*n* = 264)	Malignant (*n* = 129)	*p* Value
Demographic Characteristics				
Age, Years, median (IQR)	44.00 (35.00–54.00)	46.00 (35.00–54.00)	42.00 (34.00–51.00)	0.163
Gender, *n* (%)				
Female	297 (75.6%)	213 (80.7%)	84 (65.1%)	**0.001**
Male	96 (24.4%)	51 (19.3%)	45 (34.9%)	
Clinical Characteristics				
Thyroid Gland Type, *n* (%)				
MNG	312 (79.4%)	206 (78.0%)	106 (82.2%)	0.412
NG	81 (20.6%)	58 (22.0%)	23 (17.8%)	
Thyroid Nodule Malignancy, *n* (%)				
Yes	292 (74.3%)	185 (70.1%)	107 (82.9%)	**0.009**
No	101 (25.7%)	79 (29.9%)	22 (17.1%)	
Nodule Pathology, *n* (%)				
Malign	190 (48.3%)	110 (41.7%)	80 (62.0%)	**0.000**
Suspicious for malignancy	101 (25.7%)	74 (28.0%)	27 (20.9%)	
Follicular neoplasm	19 (4.8%)	17 (6.4%)	2 (1.6%)	
AUS/FLUS	39 (9.9%)	36 (13.6%)	3 (2.3%)	
Benign/giant nodule	11 (2.8%)	11 (4.2%)	0 (0.0%)	
ND/ND/ND	7 (1.8%)	6 (2.3%)	1 (0.8%)	
Benign	1 (0.3%)	0 (0.0%)	1 (0.8%)	
Other	5 (1.3%)	5 (1.9%)	0 (0.0%)	
ND/ND/ND/giant nodule	2 (0.5%)	2 (0.8%)	0 (0.0%)	
AUS/FLUS/giant nodule	1 (0.3%)	1 (0.4%)	0 (0.0%)	
TDMNG-benign	1 (0.3%)	1 (0.4%)	0 (0.0%)	
ND/metastatic LAP	5 (1.3%)	0 (0.0%)	5 (3.9%)	
AUS/metastatic LAP	6 (1.5%)	0 (0.0%)	6 (4.7%)	
Metastatic LAP	5 (1.3%)	1 (0.4%)	4 (3.1%)	
Surgical Characteristics				
Operation Type, *n* (%)				
Total thyroidectomy	205 (52.2%)	204 (77.3%)	1 (0.8%)	**<0.001**
TT + central LND	46 (11.7%)	23 (8.7%)	23 (17.8%)	
TT + central LND + right lateral LND	34 (8.7%)	1 (0.4%)	33 (25.6%)	
TT + central LND + left lateral LND	45 (11.5%)	0 (0.0%)	45 (34.9%)	
TT + central LND + bilateral LND	18 (4.6%)	0 (0.0%)	18 (14.0%)	
Lobectomy + completion	9 (2.3%)	9 (3.4%)	0 (0.0%)	
TT + left central LND	10 (2.5%)	7 (2.7%)	3 (2.3%)	
TT + right central LND	13 (3.3%)	8 (3.0%)	5 (3.9%)	
Lobectomy	12 (3.1%)	12 (4.5%)	0 (0.0%)	
Lobectomy, same side central and lateral dissection	1 (0.3%)	0 (0.0%)	1 (0.8%)	
Thyroid Gland Measurements				
Right lobe AP diameter, mm, median (IQR)	17.60 (15.00–21.30)	17.20 (15.00–20.90)	18.30 (15.20–22.00)	0.173
Right lobe transverse diameter, mm, median (IQR)	19.30 (16.60–23.60)	18.95 (16.48–23.60)	20.10 (17.00–23.90)	0.403
Right lobe longitudinal diameter, mm, median (IQR)	46.00 (39.70–52.00)	46.30 (39.60–52.00)	46.00 (40.50–51.90)	0.795
Left lobe AP diameter, mm, median (IQR)	15.80 (13.30–20.00)	15.65 (13.30–20.40)	15.90 (13.10–19.30)	0.971
Left lobe transverse diameter, mm, median (IQR)	18.20 (15.60–22.50)	18.25 (15.70–22.92)	17.70 (15.30–21.00)	0.080
Left lobe longitudinal diameter, mm, median (IQR)	46.00 (39.20–51.80)	46.65 (40.08–52.67)	45.10 (38.10–49.20)	**0.050**
Weight, grams, median (IQR)	22.40 (17.00–31.80)	22.00 (17.00–33.00)	24.00 (17.00–30.40)	0.754
Tumor Characteristics				
Multifocal, *n* (%)				
Yes	236 (60.1%)	146 (55.3%)	90 (69.8%)	**0.009**
No	156 (39.7%)	117 (44.3%)	39 (30.2%)	
Bilateral, *n* (%)				
Yes	168 (42.7%)	99 (37.5%)	69 (53.5%)	**0.004**
No	224 (57.0%)	164 (62.1%)	60 (46.5%)	
Tumor Subtype, *n* (%)				
PTC—Classic variant	184 (46.8%)	120 (45.5%)	64 (49.6%)	**0.001**
PTC—Follicular variant	43 (10.9%)	30 (11.4%)	13 (10.1%)	
PTC—Tall cell variant	25 (6.4%)	14 (5.3%)	11 (8.5%)	
PTC—Oncocytic variant	21 (5.3%)	14 (5.3%)	7 (5.4%)	
Uncertain malignant potential	6 (1.5%)	6 (2.3%)	0 (0.0%)	
PTC—Hobnail variant	12 (3.1%)	5 (1.9%)	7 (5.4%)	
NIFTP	19 (4.8%)	19 (7.2%)	0 (0.0%)	
PTC—Solid trabecular variant	4 (1.0%)	3 (1.1%)	1 (0.8%)	
Minimally invasive follicular Ca, Hurthle cell	1 (0.3%)	1 (0.4%)	0 (0.0%)	
Sclerosing papillary type	9 (2.3%)	1 (0.4%)	8 (6.2%)	
Classic + follicular	36 (9.2%)	23 (8.7%)	13 (10.1%)	
Warthin-like	6 (1.5%)	6 (2.3%)	0 (0.0%)	
PTC—Invasive encapsulated variant	11 (2.8%)	10 (3.8%)	1 (0.8%)	
Encapsulated follicular variant PTC	8 (2.0%)	6 (2.3%)	2 (1.6%)	
Infiltrative follicular	6 (1.5%)	4 (1.5%)	2 (1.6%)	
Differentiated high grade	1 (0.3%)	1 (0.4%)	0 (0.0%)	
Largest Tumor Diameter, mm, median (IQR)	1.20 (0.80–2.00)	1.10 (0.70–1.60)	1.50 (1.10–2.50)	**<0.001**
Lymph Node Metastasis, *n* (%)				
Yes	164 (41.7%)	37 (14.0%)	127 (98.4%)	**<0.001**
No	228 (58.0%)	226 (85.6%)	2 (1.6%)	
Number of LN Metastases, median (IQR)	4.50 (2.00–9.00)	2.00 (1.00–2.00)	6.00 (2.50–11.00)	**<0.001**
Extrathyroidal Extension, *n* (%)				
Yes	94 (23.9%)	31 (11.7%)	63 (48.8%)	**<0.001**
No	298 (75.8%)	232 (87.9%)	66 (51.2%)	
Lymphovascular Invasion, *n* (%)				
Yes	87 (22.1%)	21 (8.0%)	66 (51.2%)	**<0.001**
No	305 (77.6%)	242 (91.7%)	63 (48.8%)	

Values are presented as median (IQR) or *n* (%). LN: lymph node; TT: total thyroidectomy; LND: lymph node dissection; PTC: papillary thyroid carcinoma; NIFTP: noninvasive follicular thyroid neoplasm with papillary-like nuclear features; MNG: multinodular goiter; NG: nodular goiter; AUS: atypia of undetermined significance; FLUS: follicular lesion of undetermined significance; ND: non-diagnostic; TDMNG: toxic diffuse multinodular goiter; LAP: lymphadenopathy; Ca: cancer. Statistically significant differences (*p* < 0.05) are indicated in bold.

**Table 2:** Ultrasonographic and laboratory characteristics of lymph nodes.

Variable	Total (*n* = 605)	Benign (*n* = 380)	Malignant (*n* = 225)	*p* Value
Lymph Node Characteristics				
LN Location, *n* (%)				
Right	277 (45.8%)	167 (43.9%)	110 (48.9%)	0.261
Left	325 (53.7%)	212 (55.8%)	113 (50.2%)	
Central	3 (0.5%)	1 (0.3%)	2 (0.9%)	
Cervical Level, *n* (%)				
Level 2A	48 (7.9%)	29 (7.6%)	19 (8.4%)	**0.006**
Level 2B	42 (6.9%)	31 (8.2%)	11 (4.9%)	
Level 3	151 (25.0%)	100 (26.3%)	51 (22.7%)	
Level 4	206 (34.0%)	139 (36.6%)	67 (29.8%)	
Level 5A	5 (0.8%)	1 (0.3%)	4 (1.8%)	
Level 5B	4 (0.7%)	3 (0.8%)	1 (0.4%)	
Level 6	137 (22.6%)	74 (19.5%)	63 (28.0%)	
Level 7	11 (1.8%)	3 (0.8%)	8 (3.6%)	
LN AP Diameter, mm, median (IQR)	5.50 (4.50–7.40)	5.00 (4.00–6.00)	7.50 (5.60–10.80)	**<0.001**
LN Transverse Diameter, mm, median (IQR)	8.00 (6.10–11.20)	7.00 (5.70–9.12)	10.80 (7.60–14.20)	**<0.001**
LN Longitudinal Diameter, mm, median (IQR)	10.00 (7.40–13.60)	8.80 (6.88–11.50)	12.20 (8.90–18.00)	**<0.001**
Laboratory Findings				
TSH, mIU/L, median (IQR)	1.68 (1.16–2.64)	1.63 (1.10–2.65)	1.79 (1.23–2.58)	0.266
Anti-TPO Positivity, *n* (%)				
Yes	212 (35.0%)	154 (40.5%)	58 (25.8%)	**<0.001**
No	393 (65.0%)	226 (59.5%)	167 (74.2%)	
Anti-Tg Status, *n* (%)				
Negative	386 (63.8%)	242 (63.7%)	144 (64.0%)	1.000
Positive	219 (36.2%)	138 (36.3%)	81 (36.0%)	
Concurrent Tg, ng/mL, median (IQR)	37.90 (9.78–96.90)	31.75 (8.45–88.43)	46.00 (12.00–140.00)	0.076
Tg-Washout, ng/mL, median (IQR)	7.59 (1.18–4867.00)	2.01 (0.55–6.64)	16,141.00 (1883.00–30,000.00)	**<0.001**

Values are presented as median (interquartile range) or *n* (%). Statistically significant differences (*p* < 0.05) are indicated in bold. LN: lymph node; AP: anteroposterior; TSH: thyroid-stimulating hormone; Anti-TPO: anti-thyroid peroxidase antibody; Anti-Tg: anti-thyroglobulin antibody; Tg: thyroglobulin.

**Table 3:** Comparison of serum thyroglobulin, Tg-washout, and lymph node dimensions according to anti-Tg Status and lymph node malignancy.

	Benign	Benign	Malignant	Malignant	*p* Value	*p* Value	*p* Value	*p* Value
	Anti-Tg (−) *n* = 242	Anti-Tg (+) *n* = 138	Anti-Tg (−) *n* = 144	Anti-Tg (+) *n* = 81	a-b	c-d	a-c	b-d
Serum Tg (ng/mL)	52.50	7.09	61.05	4.75	**<0.001**	**<0.001**	**0.005**	0.487
Tg-Washout (ng/mL) (IQR)	1.81 (0.42–6.71)	2.24 (0.76–6.06)	22,327.00 (3118.5–30,000)	9872.00 (374–30,000)	0.224	**0.001**	**<0.001**	**<0.001**
LN AP Diameter (mm)	5.00	5.00	7.55	7.50	0.959	0.594	**<0.001**	**<0.001**
LN T Diameter (mm)	7.00	6.95	10.70	10.80	0.653	0.389	**<0.001**	**<0.001**
LN L Diameter (mm)	8.80	8.65	11.60	13.50	0.715	0.070	**<0.001**	**<0.001**

Data are presented as median (interquartile range). Statistical comparisons: a-b compares benign anti-Tg negative vs. benign anti-Tg positive; c-d compares malignant anti-Tg negative vs. malignant anti-Tg positive; a-c compares benign anti-Tg negative vs. malignant anti-Tg negative; b-d compares benign anti-Tg positive vs. malignant anti-Tg positive (%). Statistically significant differences (*p* < 0.05) are indicated in bold. Anti-Tg (−): anti-thyroglobulin antibody negativity; Anti-Tg (+): anti-thyroglobulin antibody positivity; Tg: thyroglobulin; LN: lymph node; AP: anteroposterior; T: transverse; L: longitudinal. Median only shown for brevity.

**Table 4:** Spearman correlation analysis between serum thyroglobulin, Tg-washout, and anti-thyroglobulin antibody.

Variable Pair	Correlation Coefficient (r)	*p* Value
Serum Tg ↔ Tg-Washout	0.260	**<0.001**
Anti-Tg ↔ Tg-Washout	−0.027	0.501
Anti-Tg ↔ Serum Tg	−0.496	**<0.001**

Spearman’s rank correlation coefficients (r) are shown with corresponding *p* values. A positive correlation indicates that as one variable increases, the other tends to increase, while a negative correlation indicates an inverse relationship. Statistically significant differences (*p* < 0.05) are indicated in bold. Tg: thyroglobulin; Anti-Tg: anti-thyroglobulin antibody.

**Table 5:** Diagnostic performance of Tg-washout for metastatic lymph node detection using Youden index-derived optimal thresholds.

Group	*n*	AUC	AUC Comparison †	Optimal Cut-Off (ng/mL)	Sensitivity %	Specificity %	PPV %	NPV %	Accuracy %
All Lymph Nodes	605	0.963	-	84.0	91.6	95.3	92.0	95.0	93.9
Anti-Tg Negative	386	0.983	Reference	84.4	96.5	94.6	91.4	97.9	95.3
Anti-Tg Positive	219	0.923	*p* = 0.008 **	65.7	84.0	96.4	93.2	91.0	91.7

Optimal cut-off values were determined using the Youden index (J = sensitivity + specificity − 1). Analyses were performed at the lymph node level. † DeLong test for comparing AUCs between anti-Tg groups. ** *p* < 0.01 indicates statistically significant difference in diagnostic performance between anti-Tg-negative and anti-Tg-positive groups. PPV: positive predictive value; NPV: negative predictive value; AUC: area under the curve; Anti-Tg: anti-thyroglobulin antibody. All performance metrics are expressed as percentages.

## Data Availability

The datasets generated and analyzed during the current study are not publicly available due to privacy and ethical concerns but are available from the corresponding author on reasonable request.

## Abbreviations

The following abbreviations are used in this manuscript:

FNA-Tg	Fine-needle aspiration thyroglobulin
DTC	Differentiated thyroid cancer
Anti-Tg	Anti-thyroglobulin antibodies
AUC	Area under the curve
FNAC	Fine-needle aspiration cytology
FNA	Fine-needle aspiration
Tg	Thyroglobulin
ATA	American Thyroid Association
IQR	Interquartile range
ROC	Receiver operating characteristic
LoD	Limit of detection
Anti-TPO	Thyroid peroxidase antibodies
LN	lymph node
TT	total thyroidectomy
LND	lymph node dissection
PTC	papillary thyroid carcinoma
NIFTP	noninvasive follicular thyroid neoplasm with papillary-like nuclear features
MNG	multinodular goiter
NG	nodular goiter
AUS	atypia of undetermined significance
FLUS	follicular lesion of undetermined significance
ND	non-diagnostic
TDMNG	toxic diffuse multinodular goiter
LAP	lymphadenopathy
Ca	Cancer
